# Identification of a novel genomic variance of BRAF1 in papillary thyroid carcinoma: A case report

**DOI:** 10.1097/MD.0000000000036978

**Published:** 2024-01-19

**Authors:** Yuguo Wang, Jian Zhao, Zhihan Tan, Jing Du, Linping Zhang, Ying Xu, Xiuying Li, Yun Cai, Hui Wang, Jianjun Jiang

**Affiliations:** a Department of Ultrasound, Nanjing Lishui District Hospital of Traditional Chinese Medicine, Nanjing, China; b Affiliated Hospital of Integrated Traditional Chinese and Western Medicine, Nanjing University of Chinese Medicine, Nanjing, Jiangsu, China; c Jiangsu Province Academy of Traditional Chinese Medicine, Jiangsu, China; d Jurong Traditional Chinese Medicine Hospital, Zhenjiang, China; e Nanjing Dian Diagnostics Group Co., Ltd, Nanjing, China; f Key Laboratory of Digital Technology in Medical Diagnostics of Zhejiang Province, Dian Diagnostics Group Co., Ltd., Hangzhou, China; g Department of Endocrinology, Yancheng City No. 6 People’s Hospital, Jiangsu, China; h Department of Ultrasound, Dafeng People’s Hospital, Jiangsu, China.

**Keywords:** BRAF, mutation, papillary thyroid carcinoma

## Abstract

**Rationale:**

Papillary thyroid carcinoma (PTC), the predominant subtypes accounting for approximately 85% of thyroid carcinomas, has a rapidly increasing global incidence rate. Statistically, approximately 74.6% PTC patients had the genomic variants of BRAF, especially BRAF^V600E^ mutation, which has been reported to stratify patients and guide clinic-therapies. However, some PTC patients may carry other nonclassical mutation patterns of BRAF, due to the complex of genomic instability. And the spectrum of BRAF mutation was not fully characterized. We reported a novel BRAF mutation pattern of PTC.

**Patient concerns:**

A 59-year-old woman was admitted to our hospital because of the slight enlargement of bilateral cervical lymph nodes in July 2023.

**Diagnosis:**

Ultrasonography revealed that the bilateral thyroid nodules of the patients both presented 1 hypoechoic nodule, which was graded as 3 of the elastic score, and the small calcification in the right lobe (Chinese-Thyroid Imaging Reporting and Data System 4c). Pathological diagnosis showed the interstitial collagen change and focal follicular epithelial papillary hyperplasia with atypical hyperplasia of the bilateral thyroid. Further puncture pathology showed that the patient had a malignant thyroid lesion with the phenotypes of papillary carcinoma and diagnosed with malignancy subsequently. Additionally, the patient harbored a novel insert on BRAF exon 15, a 6-base fragment AGACAG inserting between c.1798 and c.1799.

**Interventions:**

The patient was undergone on microwave ablation of thyroid carcinoma on July 28, 2023. After the surgery, the patient was treated on anti-infection, cold saline external application of bilateral thyroid swelling supportive treatment.

**Outcomes:**

No postoperative complications or recurrence and metastasis were found.

**Lessons:**

This is the first case of the novel nonclassical genomic variant of BRAF. Our study extends the spectrum of BRAF mutations. The patient had a favorable response to microwave ablation, indicating that in spite of the association between this mutation and high-grade malignant phenotype, this genomic variant of BRAF did not have a detrimental effect on the response of clinical treatment.

## 1. Introduction

Thyroid carcinoma is one of the most common endocrine malignancies, with a relatively favorite clinical outcomes after early diagnosis and treatment.^[1]^ Papillary thyroid carcinoma (PTC), the predominant subtypes accounting for approximately 85% of thyroid carcinomas,^[2]^ has a rapidly increasing global incidence rate,^[3]^ and was the only histological subtype that increased systematically in 25 studies countries.^[4]^

Recently, with the development and application of next-generation sequencing technology in exploration of oncogenic drivers,^[5–7]^ multiple studies reported some genomic instability leading to tumorigenesis and advancement of PTC,^[8–11]^ such as NTRK fusions^[12]^ and BRAF mutations.^[13]^ The National Comprehensive Cancer Network (NCCN) Guidelines recommended genomic testing to identify actionable mutations to assist the diagnosis and treatment.^[14]^ Notably, the genomic variants of BRAF were the most common molecular alterations in PTCs, especially BRAF^V600E^, an activating missense mutation in codon 600 of exon 15 (V600E) of BRAF gene. Although BRAF^V600E^ can be the signature molecular characteristic of PTC,^[15–18]^ the spectrum of BRAF mutation was not fully characterized. In this case, we first report a novel BRAF variant in patient with PTC. We discussed the implications of this finding for therapies and clinical outcomes.

## 2. Case presentation

A 59-year-old female was initially present with a thyroid nodule during a routine physical examination in June 2023, and had no pathological manifestation, including sore throat, retrosternal pain or choking sensation during eating, palpitation, and chest tightness or dyspnea. On July 17, 2023, the patient sough for medical advice. The initial blood parameters were as follows: white blood cell count, 4.9 × 10^9^/L; neutrophil count, 2.22 × 10^9^/L; platelet count, 294 × 10^9^/L; hemoglobin, 142 g/L; and thrombocytocrit, 0.285. The initial parameters of thyroid functions were as follows: thyroid stimulating hormone, 1.620 mIU/L; triiodothyronine, 1.63 nmol/L; free triiodothyronine, 4.26 pmol/L; free thyroxine, 15.22 pmol/L; thyroxine, 102.38 nmol/L; anti-thyroglobulin antibody < 0.90 kIU/L; and thyroid peroxidase antibody volume < 0.25 kIU/L.

**Figure 1.**
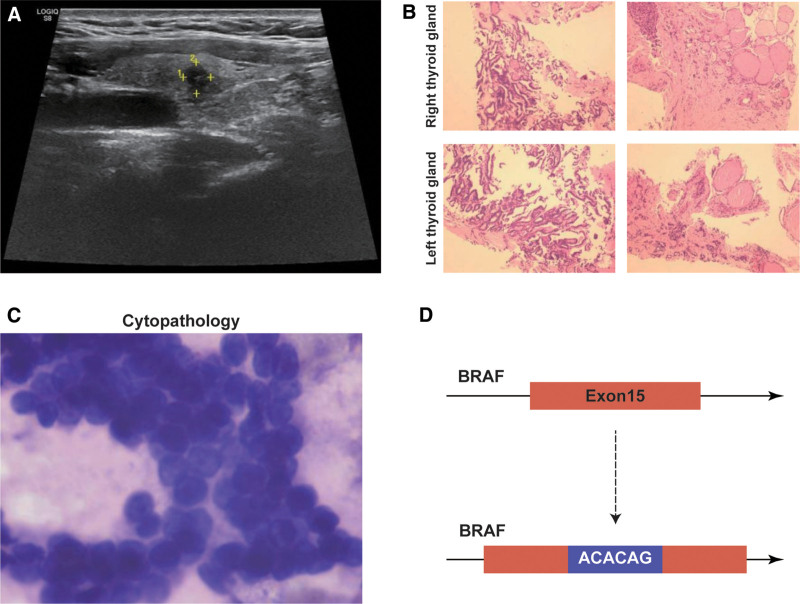
The results of examinations for the patient. (A) Results of ultrasonography. (B) Upper: pathological diagnosis of the right thyroid gland. Bottom: pathological diagnosis of the left thyroid gland. (C) Cytopathologic of the needle aspiration of the right thyroid tubercle. (D) Schematic diagram of the novel mutation of BRAF.

Ultrasonography revealed that the bilateral thyroid nodules of the patients both presented 1 hypoechoic nodule, which was graded as 3 of the elastic score, and the small calcification in the right lobe (Chinese-Thyroid Imaging Reporting and Data System 4c, Fig. 1A). Pathological diagnosis showed the interstitial collagen change and focal follicular epithelial papillary hyperplasia with atypical hyperplasia of the bilateral thyroid (Fig. 1B), suggesting the risk of carcinoma. Further puncture pathology showed that the patient had a malignant thyroid lesion with the phenotypes of papillary carcinoma (Fig. 1C) and diagnosed with malignancy subsequently. The patient reported that she had no family history of cancer.

### 2.1. Molecular genetics

For further confirmation, freshly puncture tumor tissue was used for genomic detection by Illumina sequencing platform to recognize the genomic variants of 11 genes (BRAF, HRAS, KRAS, NRAS, TP53, TERT, RET, NTRK1, NTRK3, PAX8, and THADA) associated with the thyroid carcinomas. The sequenced fragments were compared to the UCSC hg19 reference genome using STAR software. Molecular testing revealed a novel genomic variant at the exon 15 of BRAF (Fig. 1D). The bioinformatics analysis detected a 6-based fragments AGACAG was inserted between the c.1798 and c.1799 at the exon 15 of the BRAF, which was checked and visualized by Integrative Genome Viewer (IGV) (Fig. 1D). AGA encodes arginine. CAG encodes glutamine. Thus, the genomic variant resulted in the insertion of 2 extra amino acids (R and Q) between the codon 599 (599T) and 600 (600V) of exon 15, and the formation of abnormal proteins.

### 2.2. Treatment

The patient was undergone on microwave ablation of thyroid carcinoma on July 28, 2023. After the surgery, the patient was treated on anti-infection, cold saline external application of bilateral thyroid swelling supportive treatment. This patient had been followed up for 3 months, and remained free of recurrence to date, she was satisfied with the treatment.

## 3. Discussion

During the past decades, increasing evidence proved the inter- and intra-heterogeneous of solid carcinomas, which showed wide varieties of clinical presentations and influenced the therapeutic responses and outcomes of patients.^[19,20]^ Recently, with the in-depth exploration of genomic variants driving the tumorigenesis and invasiveness,^[7,21]^ molecular detection was recommended and widespread applied to recognize carcinomas, guide the clinic-therapies, and predict the prognosis.^[14,22]^

For thyroid carcinomas, BRAF mutations, involved in the tumorigenesis, invasion and metastasis, and recurrence,^[23,24]^ were highly prevalent in patients with protruding malignant thyroid nodules.^[25]^ Besides, due to the high and representative mutant frequency of BRAF^V600E^ in PTC patients (approximately 74.6%),^[18]^ American Thyroid Association and NCCN guidance recommended to detect BRAF^V600E^ mutation for diagnosis and guiding clinical therapies of thyroid carcinomas, especially the PTCs.^[14,22]^ Additionally, current risk stratification in patients with PTCs was primarily based on the BRAF^V600E^ mutation at diagnosis and influenced the treatment decision in clinical practice.^[14,22]^ For example, NCCN Guidance recommended that Dabrafenib combined with trametinib were suitable for thyroid carcinoma patients with BRAF^V600E^.^[14]^

Although BRAF^V600E^ mutation have been studies to stratify patients and guide clinic-therapies, some PTC patients could carry other nonclassical mutation patterns of BRAF, due to the complex of genomic instability. In this report, we identified a novel BRAF genomic variant in a 59-year-old female patient with PTC. To be specific, a 6-based fragments AGACAG was inserted into c.1798 and c.1799 at the exon 15 of the BRAF, leading to the insertion of 2 extra amino acids (R and Q) between the codon 599 (599T) and 600 (600V) of exon 15, and the formation of abnormal proteins. In addition, due to the insertion of 2 extra amino acids, the original codon 600 (600V) in the A-loop of the kinase domain were converted into R, activating the regulation of signal transduction from RAS to MEK inside the cell.^[26]^ Meanwhile, combined with the results of cytopathology, this nonclassical variant of BRAF might be associated with the malignancy of thyroid nodule and contribute to thyroid carcinomas.

Given of the common genomic variants of BRAF in many solid carcinomas, such as melanoma and lung cancer,^[27,28]^ the treatment of this variant in other solid tumors may have some indicative price. Some clinical trials reported that melanoma patients, carried BRAF^V600R^, achieved favorable outcomes after receiving BRAF/MEK inhibitors,^[29,30]^ indicating the potential better therapeutic responses of PTC patients carried the novel variant of BRAF after treated with BRAF/MEK inhibitors.

To the best of our knowledge, this is the first report of the novel BRAF variant in thyroid cancer. This patient had been followed up for 2 months and remained free of recurrence or metastasis to date after microwave ablation. However, since definitive conclusions cannot be made due to the nature of this case report the present findings need to be further verified in future studies.

## 4. Conclusion

In the present study, we characterized a novel genomic variant of BRAF in a 59-year-old female patient with PTC. To the best of our knowledge, this genomic variant has not previously been described or observed in PTC. The patient had a favorable response to therapy, indicating that this genomic variant of BRAF did not have a detrimental effect on the response of clinical treatment. Further studies are needed to clarify the clinical features and prognosis associated with this genomic variant.

## Acknowledgments

We would like to thank the patient and her family for their contributions and support of our research.

## Author contributions

**Conceptualization:** Hui Wang, Jianjun Jiang.

**Investigation:** Zhihan Tan, Linping Zhang.

**Methodology:** Zhihan Tan, Linping Zhang.

**Resources:** Jing Du.

**Supervision:** Jing Du, Ying Xu, Xiuying Li.

**Writing – original draft:** Yuguo Wang, Jian Zhao, Hui Wang, Jianjun Jiang.

**Writing – review & editing:** Yuguo Wang, Jian Zhao, Yun Cai, Hui Wang, Jianjun Jiang.
